# Superior detection of low-allele burden *Janus kinase 2* V617F mutation and monitoring clonal evolution in myeloproliferative neoplasms using chip-based digital PCR

**DOI:** 10.1007/s00277-024-05896-5

**Published:** 2024-07-24

**Authors:** Yiyi Lu, Lin Lin, Jiafei Lin, Beiying Wu, Gang Cai, Xuefeng Wang, Xuefei Ma

**Affiliations:** 1 Department of Laboratory Medicine, Ruijin Hospital, Shanghai Jiao Tong University School of Medicine, 197 Ruijin Er Road, Shanghai, 200025 China; 2 Shanghai Institute of Hematology, State Key Laboratory of Medical Genomics, National Research Center for Translational Medicine, Ruijin Hospital, Shanghai Jiao Tong University School of Medicine, Shanghai, 200025 China

**Keywords:** *JAK2* V617F, Myeloproliferative neoplasms, Chip-based digital PCR, Clonal evolution

## Abstract

The *JAK2* V617F is a prevalent driver mutation in Philadelphia chromosome-negative myeloproliferative neoplasms (Ph^−^MPNs), significantly affecting disease progression, immunophenotype, and patient outcomes. The World Health Organization (WHO) guidelines highlight the *JAK2* V617F mutation as one of the key diagnostic criterions for Ph^−^MPNs. In this study, we analyzed 283 MPN samples with the *JAK2* V617F mutation to assess the effectiveness of three detection technologies: chip-based digital PCR (cdPCR), real-time quantitative PCR (qPCR), and next-generation sequencing (NGS). Additionally, we investigated the relationship between *JAK2* V617F mutant allele burden (% *JAK2* V617F) and various laboratory characteristics to elucidate potential implications in MPN diagnosis. Our findings demonstrated high conformance of cdPCR with qPCR/NGS for detecting % *JAK2* V617F, but the mutant allele burdens detected by qPCR/NGS were lower than those detected by cdPCR. Moreover, the cdPCR exhibited high sensitivity with a limit of detection (LoD) of 0.08% and a limit of quantification (LoQ) of 0.2% for detecting % *JAK2* V617F in MPNs. Clinical implications were explored by correlating % *JAK2* V617F with various laboratory characteristics in MPN patients, revealing significant associations with white blood cell counts, lactate dehydrogenase levels, and particularly β2-microglobulin (β2-MG) levels. Finally, a case report illustrated the application of cdPCR in detecting low-allele burdens in a de *novo* chronic myeloid leukemia (CML) patient with a hidden *JAK2* V617F subclone, which expanded during tyrosine kinase inhibitor (TKI) treatment. Our findings underscore the superior sensitivity and accuracy of cdPCR, making it a valuable tool for early diagnosis and monitoring clonal evolution.

## Introduction

Myeloproliferative neoplasms (MPNs) are a heterogeneous group of clonal hematopoietic diseases characterized by the excessive production of one or more myeloid lineages. The World Health Organization (WHO) categorizes MPNs into two groups: Ph^+^MPN, typically referred to as chronic myeloid leukemia (CML) due to the presence of the *BCR/ABL1* fusion gene, and Ph^−^MPNs, encompassing conditions such as essential thrombocythemia (ET), polycythemia vera (PV), and primary myelofibrosis (PMF) [1, 2]. The coexistence of different types of MPNs presents a challenge for accurate diagnosis and effective treatment.

Among the various genetic mutations driving Ph^−^MPNs, the *Janus kinase 2 (JAK2)* gene mutation, particularly the p.V617F mutation, stands out as a critical factor. The *JAK2* V617F mutation, which enhances tyrosine kinase activity, is present in approximately 95% of PV cases and 50–60% of ET and PMF cases [3, 4]. Recognizing the importance of this mutation, the WHO has incorporated *JAK2* V617F detection into the diagnostic criteria for MPNs [1]. The presence of the *JAK2* V617F mutation is strongly associated with increased thrombotic risk and the severity of immunophenotype, and potential for disease progression [5–7]. Therefore, assessing the *JAK2* V617F mutant allele burden (% *JAK2* V617F) and its correlation with phenotypic markers is critical for optimizing diagnostic criteria and monitoring disease progression in MPN patients.

In general population screening, the significant underdiagnosis of possible or early MPN cases has been observed [8–10]. This underdiagnosis underscores the necessity for highly sensitive detection techniques to facilitate early diagnosis and monitor clonal hematopoietic evolution, thus preventing disease progression. The initial detection of the *JAK2* V617F mutation employed Sanger sequencing, a method with limited sensitivity for low allele burdens[3, 11–13]. In contemporary clinical practice, real-time quantitative PCR (qPCR) has become the standard method due to its higher sensitivity compared to Sanger sequencing. However, it still faces limitations such as the variability across laboratories and diverse instrumental setups, susceptibility to inhibitors, and the requirement for a standard curve to quantify mutant allele burdens [14, 15]. These challenges impact the accuracy and reproducibility of qPCR results, indicating the ongoing need to refinement of these methods and exploration of new technologies [14].The next generation sequencing (NGS) emerging as a novel technique is extensively used for the analysis of genetic variation and also evaluating the copy number of gene mutations. The sensitivity of NGS is influenced by the sequencing depth and the amount of sequence data available [16]. While clinical applications for hematological tumors, NGS sequencing depths typically range from hundreds to thousands, limiting its sensitivity for assessing low copy number variations [17–19].

Digital PCR (dPCR), including chip-based digital PCR (cdPCR) and droplet digital PCR (ddPCR), has emerged as a superior technique for precise quantification of low copy number variations due to its high sensitivity and reproducibility [20–23]. Compared to qPCR, dPCR does not require standard curves or external references. By partitioning DNA into individual chambers/droplets, dPCR significantly decreases the false negative rate by enhancing sensitivity and minimizing the probability of cross-priming and amplification of wild-type sequences [24]. This allows for the diagnosis at an early stage and more effective monitoring of treatment and minimal residual disease (MRD). ddPCR employs oil emulsion to create twenty thousand to ten million droplets, each serving as an individual PCR reaction units, offering higher sensitivity and throughput, but the droplet size was influenced by coalescence and thermo-instability, potentially causing overall error [23, 24]. Compared to ddPCR, the unified physical chamber of cdPCR decreases the variance of reaction volume, thereby improving the detection precision and accuracy. QuantStudio 3D Digital PCR system as a representative cdPCR uses a chip-based format with twenty thousand nanoscale reaction chambers of around 0.7 nL volume, each containing individual PCR amplification reactions [21, 24]. A specialized scanner detects fluorescent signals from the amplified DNA, and software calculations interpret these signals to accurately quantify the target DNA molecules [21]. Currently, some research has been investigated the effectiveness of ddPCR and qPCR in % *JAK2* V617F detection [22, 25]. However, more data is needed to directly compare the effectiveness of qPCR, cdPCR, and NGS in diagnosing MPNs and monitoring clonal evolution, and to clarify the relationship between % *JAK2* V617F and other laboratory characteristics.

In this study, we evaluated the efficacy of cdPCR in detecting % *JAK2* V617F in MPN patients, comparing its performance with qPCR and NGS. We also investigated the correlations between % *JAK2* V617F and various laboratory characteristics across different types of MPNs. Additionally, we reported a case that illustrated the clinical application of cdPCR in detecting low-allele burdens for early diagnosis and monitoring clone evolution in MPN patients.

## Materials and methods

### Patients’ samples

The clinical data of 283 de *novo* MPN patients with *JAK2* V617F mutation, but without *calreticulin* (*CARL*) and *myeloproliferative leukemia virus oncogene* (*MPL*) mutations (Table 1), and 210 wild-type control samples were retrieved from the hematology outpatient clinic at Ruijin Hospital, spanning from July 2021 to December 2022.

**Table 1:** Clinical characteristics of MPN patients with *JAK2* V617F mutation

Clinical characteristics	ET	PV	PMF
Number of patients	165	68	50
Median age, years (range)	63 (15–92)	63 (27–88)	63 (36–81)
Gender, Male/Female	71/94	45/23	24/26
Median hemoglobin, g/L (range)	141 (73–178)	181(65–235)	126 (56–175)
Median platelets, K/μL(range)	696 (282–2003)	528 (172–1775)	485.5 (33–2862)
Median white blood cell, K/μL (range)	8.7 (2.5–37.6)	11.2 (4.3–33.6)	9.4 (2.2–50.7)

*PV* polycythemia vera, *ET* essential thrombocythemia, *PMF* primary myelofibrosis

### Ethical statements

This study was approved by the Ethics Committee of Ruijin Hospital, Shanghai Jiaotong University School of Medicine (No. 2019–54) and was conducted in accordance with the Declaration of Helsinki. All procedures involving human participants were performed following the ethical standards of the institutional and national research committee.

### DNA extraction

Genomic DNA was extracted from peripheral blood samples using Magen Nucleic Acid Extraction Kit (Magen Biotechnology, China) and Thermo Fisher KingFisher Flex (Thermo Fisher Scientific, USA) based on the instructions. DeNovix Spectrophotometer (DeNovix, USA) was used to measure the absorbance of the DNA samples. The OD 260/280 ratios of DNA were range from 1.7 and 1.9 indicating a high purity of DNA.

### Chip-based digital PCR

The cdPCR was performed using a QuantStudio 3D Digital PCR system (Thermo Fisher Scientific) following the manufacturer's manual. Primers/probes mix (rs77375493, cat. no. C_101301592_10) for simultaneous detection of both the mutant and wide-type alleles were purchased from Thermo Fisher Scientific. The reaction volume was 15 μL, containing 2 μL (50 ng) of genomic DNA, 7.5 μL of Master mix, 0.5 μL of primers/probes mix (40 ×) and 5 μL of nuclease-free water. PCR assays were conducted using a ProFlex 2 × Flat PCR System (Thermo Fisher Scientific) with the following cycling conditions: 96 ℃ for 10 min, 39 cycles at 60 ℃ for 2 min and at 98 ℃ for 30 s, followed by a final extension step at 60 ℃ for 2 min. The chip used in the experiment contains 20,000 reaction chambers. For quality control (QC), at the beginning of the experiment, a QC sample run was performed using a 50% mutant allele burden of positive control. Data from each chip were analyzed using QuantStudio 3D System software to determine  the absolute copy numbers of mutant and wild-type alleles. The % *JAK2* V617F was calculated as (mutant copy number / (mutant + wild type) copy number × 100%.

### Real-time quantitative PCR

The *JAK2* V617F reference standard (Cobioer/CBP10340) derived from HEL 92.1.7 cell line was sourced from Nanjing Fukesai Biotechnology Co (Nanjing, China), with the human *JAK2* wide-type genome as the negative control. We diluted the positive mutation reference standard and determined that 12.5 ng was necessary to achieve 10,000 copies via cdPCR. Subsequently, we prepared the qPCR standards: S1 (10,000 copies/µL), followed by S2 (1,000 copies/µL), S3 (100 copies/µL), and S4 (10 copies/µL) through ten-fold serial dilutions. This method ensures the accuracy of the *JAK2* V617F mutation copy numbers in the qPCR standards used during the experiment. Primers/probes mix used in qPCR was the same with cdPCR. We conducted qPCR on an ABI 7500 device (Applied Biosystems, USA) using a 20 μL reaction mix, including 10 μL of Premix Ex Taq (Takara, China), 1.5 μL of nuclease-free water, 0.5 μL of a 40 × primers/probes mix, and 8 μL of genomic DNA (50 ng). The thermocycling conditions were 95 °C for 2 min, followed by 40 cycles of 95 °C for 10 s and 60 °C for 30 s. Mutant allele burden was calculated in accordance with the cdPCR method.

### Evaluation of the limit of detection (LoD) and limit of quantification (LoQ)

For the cdPCR method, the mean of false positive events for % *JAK2* V617F was determined in 210 wild-type control samples as limit of blank (LoB). Then, the LoD with 99% confidence was determined based on the formula: LoD = LoB + 3 × standard deviation (SD). The LoQ is defined as minimum % *JAK2* V617F that shows a significant difference from the wild-type control samples [26]. Serial dilution samples were prepared by mixing a patient sample containing a 5% mutant allele burden with wild-type DNA, resulting in % *JAK2* V617F of 5%, 1%, 0.5%, 0.2%, and 0.1%. Each diluted sample was tested in triplicate and the LoQ was established by comparing these samples with wild-type controls.

For the qPCR, the LoD was determined by preparing a series of twofold dilutions using a patient sample with a 20% mutant allele burden (validated by cdPCR) in wild-type DNA, creating *JAK2* V617F concentrations ranging from 0.3% to 20% per reaction volume. Each standard sample was analyzed in 20 replicates. The LoD allows for the detection of the minimum % *JAK2* V617F in a sample with 95% probability while ensuring a false positive rate of ≤ 5%. This was achieved by fitting a sigmoidal dose–response variable slope model using GraphPad Prism 10.

### Next generation sequencing

Libraries were generated according to the KAPA protocol provided from Roche. This process involved the careful capture of targeted genomic regions using specially designed probes, ensuring precise and accurate genomic analysis. The sequencing was conducted on Illumina NovaSeq 6000 machines, utilizing a 150-bp paired-end approach, achieving an average sequencing depth of 1500 × . The data were then processed using standard Illumina sequencing analysis procedures.

### Statistical analysis

Spearman coefficient and Bland–Altman analysis were employed to conduct an evaluation for the agreement of cdPCR with NGS or qPCR techniques. Mann-Whitney or student* t* test was used to compared the % *JAK2* V617F between the two groups. The correlations between % *JAK2* V617F and various laboratory characteristics were evaluated by Pearson or Spearman coefficients. All the aforementioned analyses were carried out using GraphPad Prism 10. ** and ***represent *P-*value < 0.01, and *P-*value < 0.001, respectively.

## Results

### Higher sensitivity of cdPCR for detecting *JAK2* V617F mutations in MPNs compared to qPCR

**Fig. 1:**
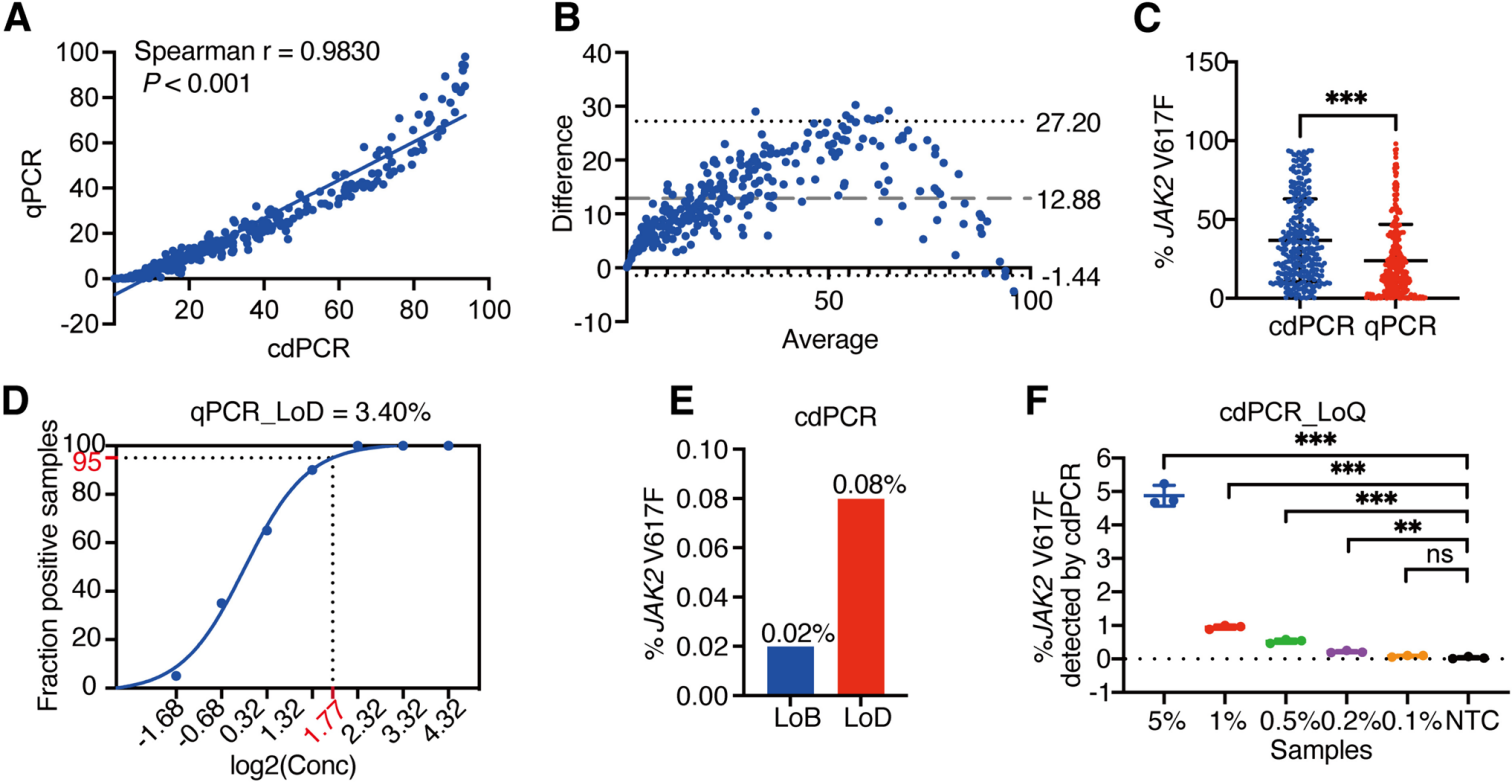
Comparative analysis of % *JAK2* V617F in MPNs using cdPCR and qPCR methods. **A** A spearman correlation scatter plot depicts the % *JAK2* V617F in MPN samples (*n* = 273), as measured by cdPCR vs. qPCR. **B **Bland–Altman plot analyzes the agreement between cdPCR and qPCR in the detection of % *JAK2* V617F in MPN samples. The plot illustrates the mean bias (central dashed line) and the 95% limits of agreement (upper and lower dashed lines indicate ± 2 SD). **C** The scatter plot displays statistically significant differences of % *JAK2* V617F measured between cdPCR and qPCR. *** represents *P*-value < 0.001. **D** The sigmoidal curve presents the fraction positive reads of 20 replicate samples at different % *JAK2* V617F (log scale) and the LoD was calculated at 95% confidence level. **E** The bar chart shows the LoB and LoD of cdPCR respectively. **F** The scatter plot presents the % *JAK2* V617F detected by cdPCR in the indicated reference samples. The LoQ was determined to be 0.2% as it was the lowest % *JAK2* V617F concentration that significantly differed from the wild-type control samples. NTC represents the negative control samples. ** and ***represent *P-*value < 0.01, and *P-*value < 0.001, respectively.

To evaluate the efficacy of cdPCR compared to qPCR in detecting the % *JAK2* V617F, we analyzed peripheral blood samples from de *novo* MPN patients carrying the *JAK2* V617F mutation without CARL and MPL mutations. The Spearman's rho test revealed a strong correlation between the results obtained via cdPCR and qPCR, with a correlation coefficient of r = 0.9830 (*P* < 0.001) (Fig. 1A). Further Bland-Altman analysis showed a bias value of 12.88 with the 95% limits of agreement ranging from -1.44 to 27.20, indicating a significant discrepancy in the % *JAK2* V617F detected by the two methods **(**Fig. 1B). The mean % *JAK2* V617F detected by qPCR (mean (SD) = 23.94% (23.03%)) was significantly lower than that detected by cdPCR (mean (SD) = 36.82% (26.28%)) **(**Fig. 1C).

To assess the sensitivity of these two methods, we determined the LoD for the lowest detectable % *JAK2* V617F. The LoD of qPCR was established at 3.40% with a 95% confidence level (Fig. 1D). For the cdPCR, we calculated the LoB as 0.02% based on the mean of false-positive events in 210 wild-type control samples, and subsequently determined the LoD as 0.08% with a 99% confidence level (LoD = LoB + 3 × SD) (Fig. 1E). These results demonstrated that cdPCR exhibited a significantly lower LoD compared to qPCR. Furthermore, we evaluated the specificity of cdPCR by analyzing the LoQ, defined as lowest % *JAK2* V617F that significantly differed from the wild-type control samples. The LoQ for cdPCR was determined to be 0.2% (Fig. 1F). These results underscored the superior sensitivity of cdPCR in detecting *JAK2* V617F mutations in MPN patients compared to qPCR.

### The cdPCR method exhibits a higher positive detection rate for *JAK2* V617F than NGS

**Fig. 2:**
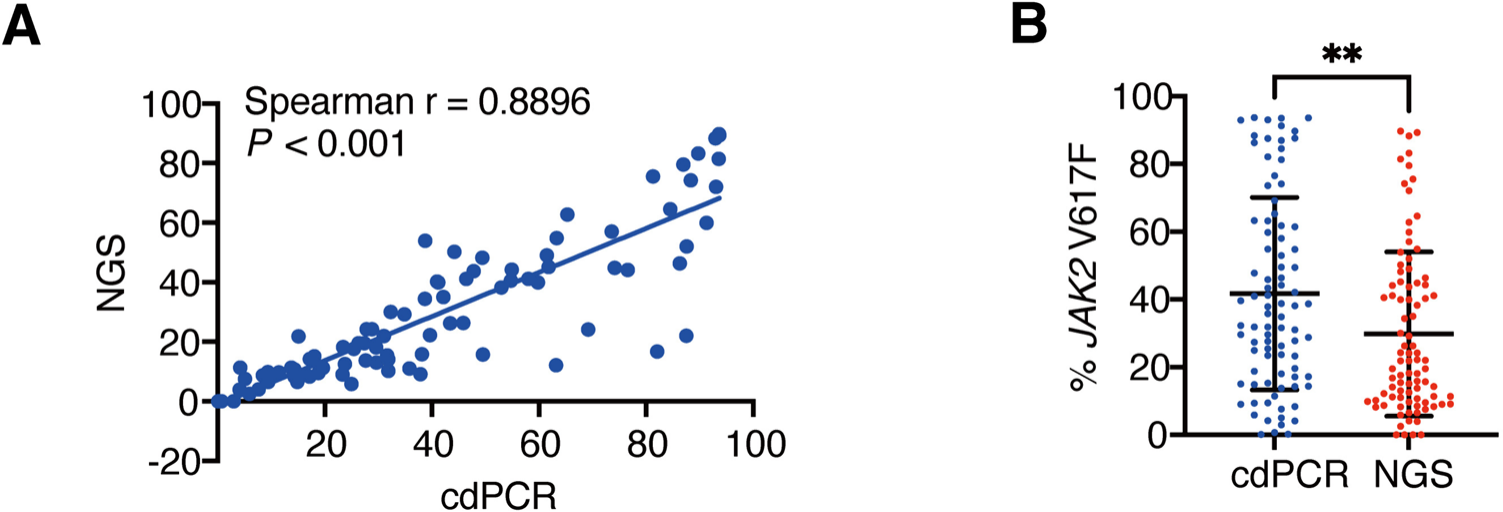
Correlation between cdPCR and NGS in detecting % *JAK2* V617F. **A** The correlation between the % *JAK2* V617F as detected by cdPCR (x-axis) and next-generation sequencing (NGS) (y-axis) is represented by a spearman correlation scatter plot. **B** The scatter plot displays statistically significant differences of % *JAK2* V617F measured between cdPCR and NGS. ** represents *P*-value < 0.01.

Following the observed superior efficacy and sensitivity of cdPCR over qPCR, we compared cdPCR with NGS techniques using 88 MPN patient samples. The correlation of % *JAK2* V617F quantification between NGS (at an average depth of 1500 ×) and cdPCR was established, with a Spearman correlation coefficient of r = 0.8896 (*P* < 0.001) (Fig. 2A**)**. However, a discordance rate of 4.5% (4 out of 88 samples) was observed between the two methods (Table 2). The four samples that NGS failed to detect were identified by cdPCR with mutant allele burdens below 3%, possibly due to the limited sequencing depth of NGS. Additionally, NGS failed to detect one sample that was positive by qPCR, and three samples identified with the *JAK2* V617F mutation by cdPCR were not detected by either NGS or qPCR (Table 2).

**Table 2:** Comparison of NGS with cdPCR and qPCR methods in MPN patients

NGS	cdPCR		qPCR	
	Positive	Negative	Positive	Negative
Positive	84	0	82	0
Negative	4	0	1	3
Total	88	0	83	3

Positive sample: A sample was classified as “Positive” when the % *JAK2* V617F was detected at any level above 0% by the respective method

Negative sample: A sample was considered “Negative” if the *JAK2* V617F mutation was not detected by the methods

A comparative analysis of mutant allele burden revealed that the average % *JAK2* V617F determined by NGS (mean (SD) = 29.79% (24.26)) was lower than that determined by cdPCR (mean (SD) = 41.71% (28.41)) (*P* < 0.01) (Fig. 2B). These results demonstrated that cdPCR exhibited higher sensitivity and accuracy in detecting *JAK2* V617F mutations compared to NGS. The cdPCR proved particularly well-suited for clinical applications, effectively minimizing the probability of missed detection in MPN samples with low-allele burden mutations.

### Correlations between % *JAK2* V617F and various laboratory characteristics in MPNs

To explore the advantages of cdPCR in detecting *JAK2* V617F mutations and their clinical implications, we further investigated the % *JAK2* V617F in peripheral blood cells from MPN patients. The % *JAK2* V617F was notably higher in patients with PV (mean (SD) = 52.11% (25.46)) and PMF (mean (SD) = 51.20% (27.16)) compared to those with ET (mean (SD) = 26.60% (21.08)) (Table 3). Correlations between % *JAK2* V617F and laboratory characteristics presented differences among three types of MPNs (Table 3). First, positive correlations were observed between % *JAK2* V617F and the white blood cells (WBC) counts (*P* < 0.05), as well as the endogenous serum globulin beta-2 microglobulin (β2-MG) levels (*P* < 0.001) across all three types of MPN patients. Moreover, elevated % *JAK2* V617F was associated with higher lactate dehydrogenase (LDH) levels in three MPN subtypes (*P* < 0.05). Second, specific correlations were noted only in PV and PMF: in PV, a positive correlation was found between hemoglobin (*P* = 0.02) and % *JAK2* V617F, while in PMF, a positive correlation was seen with erythropoietin (EPO) levels (*P* = 0.02). Notably, PV patients (median: 181 g/L, range: 65–235 g/L) exhibited higher median hemoglobin levels compared to those with ET (median: 141 g/L, range: 73–178 g/L) and PMF (median: 126 g/L, range: 56–175 g/L) (Table 1). No significant correlation was found between serum ferritin levels and % *JAK2* V617F in three types of MPNs (*P* > 0.05). Third, a positive correlation emerged between platelet counts and % *JAK2* V617F in ET patients (r = 0.2115, *P* = 0.006), while a negative correlation was seen in PMF patients (r = -0.2893, *P* = 0.04) (Table 3). The median platelet counts were higher in ET than in PV and PMF (Table 1**)**.

**Table 3:** Correlation between laboratory characteristics and *JAK2* V617F allele burden in MPN patients

Laboratory characteristics	ET (% *JAK2* V617F: 26.60 ± 21.08)		PV (% *JAK2* V617F: 52.11 ± 25.46)		PMF (% *JAK2* V617F: 51.20 ± 27.16)	
	r	*P*-value	r	*P*-value	r	*P*-value
White blood cell	0.3722	< 0.001	0.5471	< 0.001	0.3615	0.01
β2-MG	**0.4915**	< 0.001	**0.6433**	< 0.001	0.5844	< 0.001
LDH	0.4805	< 0.001	0.4166	0.02	**0.6766**	< 0.001
Hemoglobin	-0.0316	0.69	0.2863	0.02	-0.2106	0.14
EPO	-0.0028	0.98	0.1169	0.52	0.3601	0.02
Serum ferritin	0.1835	0.15	-0.3065	0.12	0.1512	0.33
Platelets	0.2115	0.006	-0.1642	0.18	-0.2893	0.04

% *JAK2* V617F indicates the mutant allele burden of *JAK2* V617F and is presented as means ± standard deviation

Notably, β2-MG levels exhibited a stronger correlation with % *JAK2* V617F compared to other clinical indicators, including WBC, LDH, hemoglobin, EPO, serum ferritin, and platelet counts, in both ET (r = 0.4915, *P* < 0.001) and PV (r = 0.6433, *P* < 0.001). In PMF patients as well, β2-MG (r = 0.5844, *P* < 0.001) showed significant relevance with % *JAK2* V617F, ranking second only to LDH (r = 0.6766, *P* < 0.001) (Table 3). Given that % *JAK2* V617F levels correlate with MPN severity, these findings provided evidence for the clinical implications of β2-MG in MPN progression.

### Case report on the performance of cdPCR in detecting low-allele burden *JAK2* V617F mutation

**Fig. 3:**
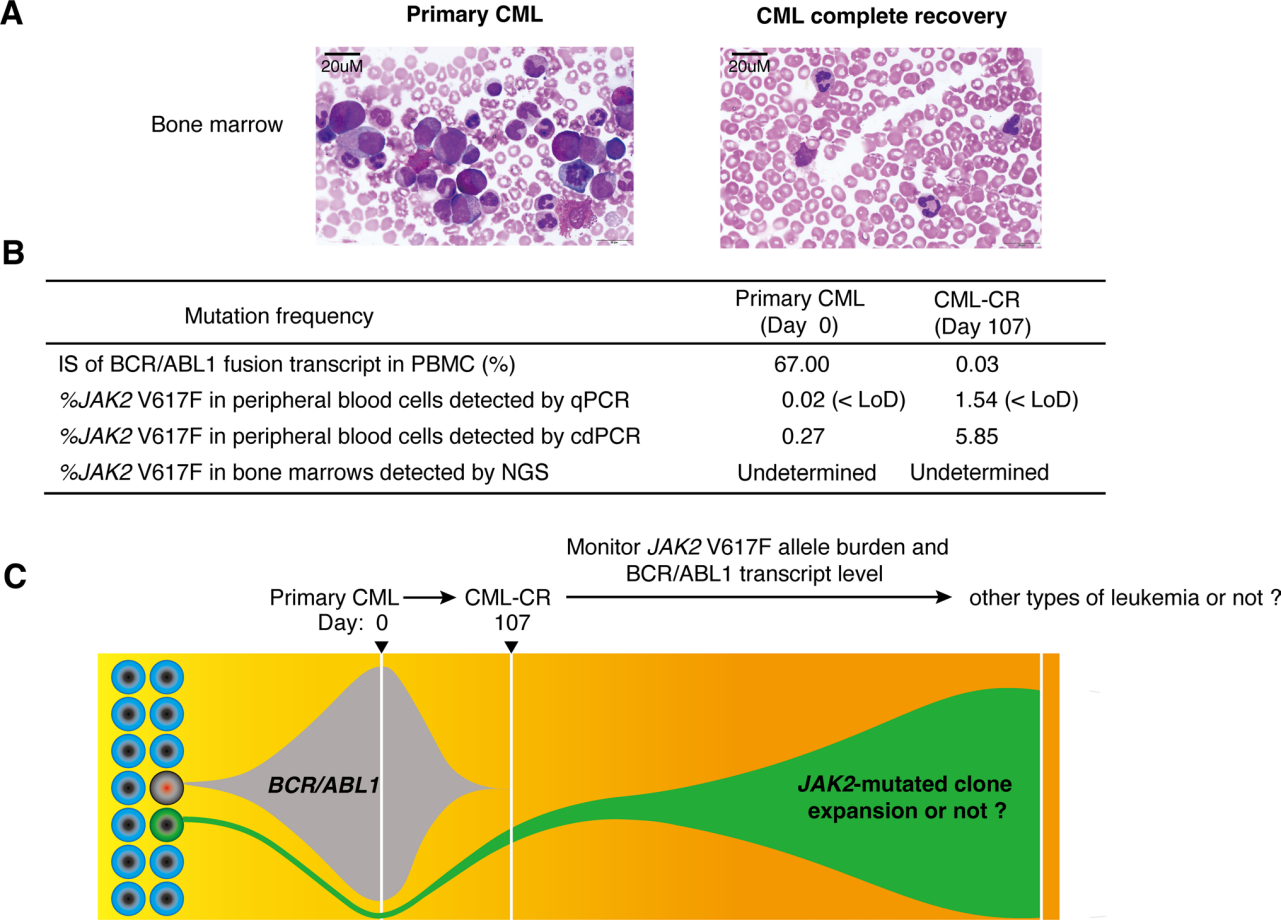
A case report of clonal evolution in CML. **A** Bone marrow biopsies images present the primary and complete recovery CML (CML-CR) phases, respectively. **B** The table shows the IS of BCR/ABL1 fusion transcript and % *JAK2* V617F detected by qPCR, cdPCR or NGS in the primary CML and CML-CR phases, respectively. **C** A model depicts that identifying de *novo* clones and monitoring % *JAK2* V617F in CML patients with *BCR/ABL1* and *JAK2* V617F dual clones by cdPCR, could be vital for promptly monitoring of clonal evolution and disease progression from CML to other types of leukemia

The cdPCR has proven to be highly effective for discovering novel clones and monitoring clonal evolution in MPNs, particularly for low-allele burden mutations that fall within the detection “grey-zone” where qPCR and NGS cannot reliably quantify small mutation clones in clinical application. In this case, we observed a 54-year-old man who initially diagnosed with CML, carrying the *BCR/ABL1* mutation along with a small *JAK2* V617F clone hidden within primary CML. After 107 days of tyrosine kinase inhibitor (TKI) Flumatinib treatment, the patient achieved complete remission (CML-CR) (Fig. 3A). The international scale (IS) of the *BCR/ABL1* fusion transcript in peripheral blood mononuclear cells (PBMCs) decreased from 67% to 0.03%, accompanied by expansion of the % *JAK2* V617F clone. The small *JAK2* V617F mutation clone was identified by cdPCR from the primary CML to CML-CR stage, with the mutant allele burden increasing from 0.27% to 5.85% (Fig. 3B).

We also used qPCR and NGS to validate our findings. Although qPCR detected the *JAK2* V617F mutation in primary CML (0.02%) and CML-CR (1.54%) stages, the mutation burden detected is significantly below the qPCR sensitivity threshold of 3.40% (LoD) and lower compared to the values detected by cdPCR. The NGS assay did not detect the % *JAK2* V617F in either the primary CML or CML-CR stages in the bone marrow sample (Fig. 3B). These findings highlighted the importance of using cdPCR in screening low-allele burden mutations in early MPN patients, monitoring clonal evolution, and distinguishing whether the clones are original or induced by drug pressure (Fig. 3C).

## Discussion

Our study has showcased the precision and sensitivity of cdPCR as a diagnostic method for monitoring low-allele burden of *JAK2* V617F in MPNs, surpassing the capabilities of qPCR and NGS in clinical applications. Moreover, we conducted an in-depth analysis of the relationship between % *JAK2* V617F and various laboratory characteristics, especially emphasized the significant correlation between β2-MG levels and % *JAK2* V617F. Finally, we reported a case that illustrated the application of cdPCR in detecting low-allele burdens in a de *novo* chronic myeloid leukemia (CML) patient with a hidden *JAK2* V617F subclone, which expanded during TKI treatment.

The findings of this study revealed high concordance between cdPCR, qPCR, and NGS in detecting the % *JAK2* V617F. Importantly, cdPCR demonstrated significantly higher sensitivity than both qPCR and NGS. This aligns with previous research by E. KINZ, which corroborated the conformance between cdPCR and qPCR methods, and affirmed the accuracy of cdPCR in detecting % *JAK2* V617F [20]. Notable differences between qPCR and cdPCR may include the higher probability of cross-priming and increased wild-type background signal in qPCR, potentially lower amplification efficiency of qPCR enzymes, increased susceptibility of qPCR to inhibitors, and variations in hardware performance. Discrepancies between NGS and cdPCR are likely resulting from the insufficient sequencing depth in NGS (1500 ×), which may lead to underestimation of allele burden compared to cdPCR, and biases introduced during sample preparation, such as capture and amplification biases and single-stranded DNA bias. Overall, these findings underscore the robustness and higher sensitivity of cdPCR in detecting % *JAK2* V617F, reinforcing the reliability and accuracy of cdPCR in both clinical and research settings.

The LoD in our methodologies was influenced by several factors. For the qPCR, we observed a higher LoD compared to other publications. This discrepancy can be attributed to various reasons. First, the type and quality of enzymes used in qPCR reactions in our study may differ from those used in studies by Link-Lenczowska and La Rocca [22, 25], resulting in variations in amplification efficiency and specificity. Second, differences in reaction volumes and DNA input amounts between our study and others can significantly impact qPCR sensitivity. Third, variations in probe design and binding efficiency lead to significant differences in detection outcomes. Probe specificity and affinity for the target sequence are essential for accurate quantification. Fourth, increasing the number of replicates enhances accuracy and reliability by reducing variability and improving the detection of low-abundance targets. Fifth, instrument performance, such as optical calibration and noise, can affect the detection results. For the cdPCR, the LoD (0.08%) in our study is close to the previous study (0.1%) [20]. However, compared to ddPCR, the LoD of cdPCR appears higher than ddPCR because ddPCR utilizes droplet systems that can generate up to 10 million partitions, thereby offering higher sensitivity and throughput [24].

Our results emphasized the correlations between % *JAK2* V617F and various laboratory characteristics, particularly β2-MG levels. Evidence indicates that the % *JAK2* V617F correlates with the severity of immunophenotype as well as the progression of MPNs [5, 6]. Recent large-scale expression profilings utilizing single-cell RNA sequencing (scRNA-seq) and microarray data have shown significant upregulation of HLA I and β2-MG levels in patients with *JAK2* V617F mutations [27, 28]. While these studies provide insights into the relationship between % *JAK2* V617F and β2-MG, direct correlation analysis is limited. Our study specifically revealed that β2-MG levels showed a strong correlation with % *JAK2* V617F compared to other indicators in ET and PV. These findings provide new insights into the clinical implications of β2-MG in MPN progression and immune-inflammatory risk, and also offer potential clues for investigating its underlying mechanisms in the future.

Utilizing cdPCR for MPN diagnostics substantially improves early detection, screening at-risk individuals, treatment assessment, and monitoring of MRD. Although WHO includes *JAK2* V617F mutation as a criterion for MPN diagnosis, it may not specify the allele burden range [29]. The Danish General Suburban Population Study (GESUS) showed that 42% of mutation-positive non-MPN patients had elevated blood cell counts, with a median allele burden of 0.14%, suggesting that individuals with driver gene mutations, even with low mutation burdens, should be considered at risk for MPNs [9]. Therefore, these researchers proposed a baseline screening strategy that includes detecting either *JAK2* V617F or *CALR* mutations along with elevated blood cell counts to enhance early MPN detection sensitivity [10]. Continuous monitoring of allele burden is recommended for distinguishing early MPNs from non-MPNs in individuals with allele burdens below 1% [10]. Additionally, cdPCR proved sensitivity in assessing treatment responses and predicting outcomes, such as evaluating interferon-alpha2 (IFN-alpha2) therapy effectiveness through accurate % *JAK2* V617F measurement to determine major molecular response (MMR) (below 1%) [30], and predicting prognosis and recurrence by monitoring post-transplantation MRD levels, with % *JAK2* V617F above 1% indicating higher risk[31–33]. Although qPCR can theoretically detect as low as 0.12% and NGS below 0.01%, achieving this sensitivity in clinical practice is limited by practical challenges such as sample quality and dilution control [22, 34]. Therefore, cdPCR provides a more reliable and sensitive alternative, improving accuracy in detecting low mutation burdens, and overcoming the limitations of qPCR and NGS in the "grey zone" of *JAK2* V617F detection.

Investigating clonal evolution by cdPCR could yield valuable insights into clonal by potentially revealing alterations in the genetic makeup of persistent cancer cells over time. Using cdPCR, we identified a low-allele burden of *JAK2* V617F subclone in a de *novo* CML patient, which expanded during TKI treatment, highlighting the potential of cdPCR in uncovering these hidden subclones. This technology's LoD of 0.08% allows for early identification of mutations that may be undetermined by qPCR and NGS methods, crucial for predicting disease progression and tailoring therapeutic strategies. Recently, it has been reported that less than 1% of all MPN cases present concomitant both *BCR/ABL1* and *JAK2* V617F mutations [35]. Some viewpoints suggested that the second Ph^−^ MPN was initially concealed and only became noticeable following treatment with TKIs [13, 36]. However, these conclusions were only based on the detection of *JAK2* V617F mutations using qPCR, allelic discrimination assay or DNA sequencing. With the emergence of dPCR technology and the improvement of detection sensitivity, clinicians can distinguish whether the *JAK2* V617F mutation is a hidden subclone within CML or a novel mutation induced by drug pressure. Future research will focus on elucidating the mechanisms behind subclone expansion and exploring the therapeutic implications of early mutation detection.

## Data Availability

No datasets were generated or analysed during the current study.
